# miR-140-3p enhances the sensitivity of LUAD cells to antitumor agents by targeting the ADAM10/Notch pathway

**DOI:** 10.7150/jca.78835

**Published:** 2022-11-21

**Authors:** Hao Meng, Bo Li, Wei Xu, Renquan Ding, Shiguang Xu, Qiong Wu, Yingshi Zhang

**Affiliations:** 1 Department of Thoracic Surgery, General Hospital of Northern Theater Command, Shenyang City 110011, Liaoning Province, China.; 2 Department of Clinical Pharmacy, Shenyang Pharmaceutical University, Shenyang City 110011, Liaoning Province, China.

**Keywords:** Notch pathway, A disintegrin and metalloproteinase domain 10, miR-140-3p, chemotherapeutic agents, molecular targeted agents

## Abstract

**Background:** The Notch pathway, which is related to the drug-resistance of lung adenocarcinoma (LUAD) type of non-small cell lung cancer (NSCLC) cells, is activated by cleavage of Notch proteins mediated by ADAMs, ADAM10 or ADAM17.

**Methods:** In the present study, our results demonstrated that of these two ADAMs, the expression of ADAM10 in clinical samples of the LUAD type of NSCLC was much higher than that of ADAM17, while miR-140-3p - an miRNA that could target ADAM10 - was identified by an online tool: miRDB (miRNA database). The detail function and mechanism of miR-140-3p in regulating the sensitivity of NSCLC cells to antitumor drugs was systematically explored *in vitro* and *in vivo*.

**Results:** In A549, a typical NSCLC LUAD cell line, miR-140-3p decreased ADAM10 expression and repressed activation of the Notch pathway by repressing cleavage of Notch proteins. The expression of miR-140-3p was negatively related to ADAM10 in clinical specimens. Nucleocytoplasmic separation/subfraction assays showed that miR-140-3p was able to inhibit the cleavage of Notch protein, and led to the accumulation of Notch intracellular domains (NICD) in the nucleus. Overexpression of miR-140-3p enhanced the sensitivity of A549 cells to antitumor agents by targeting the 3'UTR region of ADAM10 mRNA in both cultured cells and *in vivo* models.

**Conclusion:** ADAM10 plays a major role in LUAD, and miR-140-3p acts on ADAM10 and inhibits its expression and the cleavage of Notch protein, leading to the inhibition the activity of the Notch pathway, and ultimately upregulating LUAD cell sensitivity to anti- tumor drugs.

## Introduction

Lung cancer poses a serious threat to the health of individuals worldwide and in China, and its morbidity and mortality rank first among all malignant tumors 1,2. Among the types of lung cancer, non-small cell lung cancer (NSCLC) is the most important and common pathological type 3-5. Furthermore, NSCLC is the most important type of lung adenocarcinoma, making lung adenocarcinoma-related research important 3-6. Currently, antitumor drug treatments for lung adenocarcinoma mainly include various small molecule protein kinase inhibitors and cytotoxic chemotherapeutics 7-12. Although these drugs or drug combinations are believed to prolong the lives of patients and improve their quality of life, there are still many shortcomings: (1) the sensitivity of patients to these antitumor drugs is purely individual 13-15; (2) antitumor drug treatment is prone to drug resistance 13-15; and (3) the side effects of antitumor drug treatment are very serious 16. Small molecule inhibitors or therapeutic antibodies targeting the PD-1/PD-L1 pathway have been available for a short time, and the influencing factors or biomarkers that influence the efficacy of these drugs are not yet clear 17-19. Therefore, it is of great significance to study and develop safer and more effective anti-adenocarcinoma drug treatment strategies, to achieve the superior treatment strategies at drug treatment doses that are the same or smaller.

Increasing evidence has shown that the Notch pathway is not only an important positive regulator of NSCLC but also plays a central role in antitumor drug resistance 20-22. It has been confirmed that in the process of anti-tumor treatment, chemotherapy drugs and radiotherapy do more harm than good to the body, but they are serious damaging factors for malignant tumor cells/tissues by targeting tumor cell components or to macromolecules 23,24. In this process, the Notch pathway can be activated 23,24. The activated Notch pathway can induce cell survival, anti-apoptosis-related factors, and malignant tumor cell epithelial-mesenchymal transition (EMT) 25. In this way, malignant tumor cells respond to exogenous damage factors (i.e., stress factors) through Notch, which is involved in the cell's stress response mechanism to improve cell resistance, promote survival, and anti-apoptosis mechanisms, ultimately resulting in cell resistance to these antitumor treatments 22-25. In this process, the activation of the Notch pathway is mainly dependent on the cleavage of the Notch protein: when the Notch protein is cleaved, the extracellular and intracellular segments of the Notch protein separate to release the intracellular products, which migrate into the nucleus and ultimately mediate the expression of factors related to cell survival and anti-apoptosis 25,26. The activation of Notch protein is a two-step cleavage, process in which the first cleavage is completed by two ADAMs: ADAM17 and ADAM10 27-29. This makes ADAM17 or ADAM10 an ideal intervention target for the treatment of malignant tumors. It is of great significance to determine the expression of both in different tumor tissues, and to develop corresponding intervention strategies.

MicroRNA is a type of small noncoding RNA that can recognize and act on the 3'UTR of the target gene mRNA in a sequence-specific manner to degrade the mRNA and finally achieve post-transcriptional silencing 30-32. Not only can miRNA be used as an important strategy for anti-tumor therapy but also the lack of expression of miRNA is also an important mechanism for the higher expression of pro-oncogenes in malignant tumor tissues 33. In this study, the expression of two ADAMs in lung adenocarcinoma (LUAD) tissues was first determined, and it was found that ADAM10 is the main ADAM subtype that plays a major role in LUAD. IT was found that miR-140-3p is the miRNA that acts on ADAM10, miR-140-3p can inhibit the activity of the Notch pathway by acting on ADAM10, and ultimately up-regulates the sensitivity of lung adenocarcinoma cells to anti-tumor drugs.

## Materials and Methods

### Clinical specimens, cells lines, and agents

A total of 26 pairs of malignant tumor tissues and adjacent non-tumor tissues were collected and stored by the Department of Thoracic Surgery, General Hospital of Northern Theater Command from November 2019 to March 2021. All clinical specimens were obtained from routine surgical resection and LUAD was confirmed by pathological analysis by the General Hospital of Northern Theater Command) of the author's institution. The diagnosis, inclusion and exclusion criteria of patients were all in accordance with the clinical diagnosis and treatment guidelines for lung cancer of the Chinese Medical Association (2022 version) 34. The patient was diagnosed according to imaging methods, including CT (Computed Tomography), MRI (Nuclear Magnetic Resonance Imaging), or PET [Positron Emission Computed Tomography]. Head CT, bone CT, and liver-gallbladder-pancreas-kidney-adrenal-color ultrasound were performed before surgery. The patients with no severe obstructive difficulty in pulmonary function; no serious heart disease (including no stent placement within half a year, and cardiac function>56%) underwent surgical operation. The pathological staging and classification of the specimens removed by surgery are determined by pathological staining and analysis (that is, confirmed as lung adenocarcinoma). The baseline information of the patients were shown as Table 1. The sample size (26 paired nontumor/tumor specimens) has adequate power to detect a pre-specified effect size (the 1-β: 0.8; α/2: 0.025; P < 0.05) of the statistical analysis by original hypothesis/ alternative hypothesis. The original hypothesis was that the expression level of the targeting gene (for example, ADAM10 or miR-140-3p) was not significantly different in the non-tumor tissues compared with the tumor tissues; whereas the alternative hypothesis was that the expression level of the targeting gene was significantly different in the non-tumor tissue compared with the tumor tissue.

The expression vector containing full length sequences of ADAM10 (the wild type of ADAM10 or the ADAM10 with the mutated miR0140-3p targeted sites located in its 3' untranslated region (3'UTR), hsa-pre-miR-140 or NICD was constructed and prepared as lentivirus and stored at our lab as described in previous studies 25,35-37. The 3'UTR region sequence of ADAM10 containing wild-type or mutant miR-140-3p action site (about 200bp upstream and downstream of the action site) was cloned into the pGL4.26 vectors to form the luciferase reporters named as Luc-1 or Luc-1^Mut^.

The NSCLC LUAD cell line A549, NSCLC large cell lung cancer (LCC) cell line H460 (NCI-H460), and NSCLC Lung squamous cell carcinoma (LSCC) cell line H520 (NCI-H520), and WI-38, a non-tumor lung derived cell line was purchased from the Type Culture Collection of the Chinese Academy of Sciences (the culture collection center of the Chinese government, Shanghai, People's Republic of China).

The antitumor agents, gemcitabine, paclitaxel, etoposide, doxorubicin, gefitinib (Cat. No.: S1025), erlotinib (Cat. No.: S7786), osimertinib (Cat. No.: S7297) or anlotinib (Cat. No.: S8726), were purchased from Selleck Corporation, Houston, TX, USA. For *in vitro* cell-based cell survival assays or MTT experiments. The antitumor agents (the powders of agents with purity > 98.7%) were carefully dissolved by using the organic solvents, dimethyl sulfoxide (DMSO), and then diluted by DMEM without FBS with stirring and ultrasonic solubilization 38-41. The concentrations of these agents used in this study in DMSO or DMEM are listed in Table 2. For the subcutaneous tumor experiments in nude mice, gefitinib (4 mg) was dissolved by a mixture of organic solvents: DMSO (15 μL), PEG400 (polyethylene glycol) (60 μL), and Tween80 (40 μL) and was carefully added with physiological saline to a total volume 2 mL with stirring and ultrasonic solubilization 38-41. This Gefitinib-containing solution was used for oral administration to nude mice.

### Antibodies and Western blotting

Antibodies against ADAM10 (Cat. no ab124695), Lamin A (Cat. no ab108595), GAPDH (Cat. no ab8245) and β-actin (Cat. no ab205) were obtained from Abcam (Cambridge, MA, USA). Antibodies to detect the NICD of Notch protein was generated by our lab as described in previous studies 42-44. A549 cells, following transfection with vectors, were harvested for total protein extraction for western blotting experiments under standard protocols. The cellular sub-fraction assays were performed following the methods described in previous studies 42-44. Briefly, the A549 cells were transfected with plasmids and then trypsinized and harvested. The cells were disrupted using sonication, and the nuclear fraction was collected at 800 rpm, while the cytoplasmic fraction was collected after centrifugation at 12000 rpm. The above components were detected using western blotting. Lamin A was used as an to normalize nuclear components/sub-fraction, while β-Actin was used for cytoplasmic components/sub-fraction. The images of western blot were quantitatively analyzed by Image J software (National Institutes of Health [NIH], Bethesda, Maryland, USA) 45-47. The total area and the intensity of bands of ADAM10, NICD or loading control (Lamin A for nuclear sub-fraction and β-Actin for cytoplasm sub-fraction) from in the western blot images was quantitatively identified by Image J. The expression level of ADAM10 or NICD was adjusted by β-Actin or Lamin A 45-47.

### Cell culture and proliferation analysis

The MTT assay was used to evaluate the survival-inhibitory effect of antitumor drugs on A549 cells as following the methods described in previous studies 45-48. Briefly, transfected A549 cells were seeded in 96-well plates (8000 cells per well) and then treated with the indicated concentrations of antitumor agents for 48h. The cells were then subjected to MTT assay agents for 5-6 h. Lastly, the cell samples were diluted using SDS, the absorbance was read using a multi-function microplate reader at 490 nm. The inhibition rates of the drug on A549 cells was determined bas on the 490 nm values, and a drug inhibition curve was constructed for the A549 cells, and the IC_50_ value of the drug effect was determined.

### Quantitative polymerase chain reaction (qPCR) and luciferase assays

Quantitative polymerase chain reaction experiments were carried out according to the methods reported in previous studies and in accordance with the manufacturer's instructions 49-51. Briefly, total RNA samples from A549 and other cells and tissue samples were extracted and then reverse transcribed into cDNA. The ABI-7500 real-time quantitative PCR instrument was used for qPCR experiments, and GAPDH was used as the internal reference to calculate the expression of each gene in each sample. Finally, the relative expression of each gene in each group was determined based on the control group (fold change of control), and a heatmap was drawn 51. The primers of ADAM10 were Forward Sequence, 5'-GAGGAGTGTACGTGTGCCAGTT-3' and Reverse Sequence, 5'-GACCACTGAAGTGCCTACTCCA-3'. The primers of hsa-pre-miR-140-3p were: (1) RT-primer, 5'-GTCGTATCCAGTGCGTGTCGTGGAGTCGGCAATTGCACTGGATACGACCCGTG-3', the Forward Sequence, 5'-AGAACCACGGGTCGTATCCA and Reverse Sequence, 5'-CAGTGCGTGTCGTGGAGT-3. Other primers were gifts from Dr. Yan Ma in Department of Gastroenterology and Hepatology, Chinese PLA General Hospital, Beijing, China 35.

For the luciferase assays, A549 cells were co-transfected with the miRNA-140-3p, Luc-1 or Luc-1^Mut^ and harvested for luciferase assays according to the previous publication 30. Luciferase test kit produced by Promega Company was used for the experiment. After the cells were scraped off with a scraper, the cells were mixed with the lysate, and then the cells were broken by shaking (vortex shaking for 15 min). After that, the cells were centrifuged at 4 °C at 12000 rpm for 10 min, and the supernatant was collected as the cell sample. Luciferase activity and β-Gal activity to β-Gal activity reading corrects luciferase reading. Relative luciferase activation (folds to control) of each group is determined with luciferase activity of control group as unit 1.

### Subcutaneous LUAD tumor model

The *in vivo* antitumor activity of agents on A549 cells was examined using the subcutaneous tumor model 52,53. The use of nude mice and the relevant protocols were reviewed and approved by the Institutional Animal Care and Use Committee of the General Hospital of Northern Theater Command, People's Liberation Army of China. The subcutaneous tumor model was performed according to the methods described in previous studies. A549, previously transfected with plasmids/vectors, were injected into nude mice (approximately 5×10^6^ cells per injection point). The mice were administered the indicated doses of gefitinib via oral administration. The length and width of tumor tissues were measured using a Vernier caliper and the tumor volume was calculated as tumor width × tumor width × tumor length / 2. Tumor tissue was accurately weighed using a precision balance.

### Ethics statement

The collection, and usage of clinical specimens, and all the methods and experimental design/protocol were reviewed and approved by the ethics review organization: the Medical Ethics Committee of General Hospital of Northern Theater Command. All experiments were performed according to the Helsinki Declaration (WHO) with the written consent from patients.

The animal experiments related information (including animal breeding, animal welfare, experimental design and methods of animal experiments) were all reviewed and approved by the Animal Ethics Committee of General Hospital of Northern Theater Command. All experiments were performed in accordance with the UK Animals (Scientific Procedures) Act, 1986 and associated guidelines.

### Statistical analysis

Statistical analyses in the present work were performed by Bonferroni's correction with two-way ANOVA using SPSS Software (software version 9.0, IBM Corporation, Armonk, NY, USA). The IC_50_ values were calculated using an Origin software (Version No 6.1, OriginLab Corporation, Northampton, MA, USA). A P-value of <0.05 was considered to indicate statistically significant differences between two groups.

## Results

### The expression of ADAM10 was much higher compared with ADAM17 in LUAD clinical specimens

**Figure 1:**
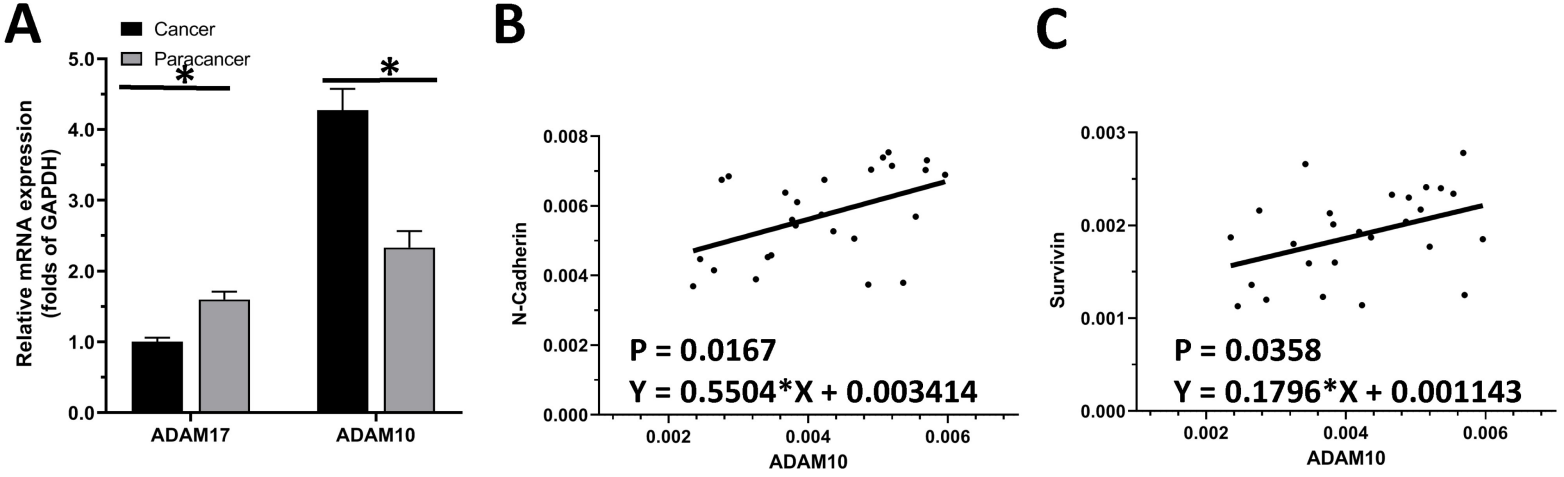
** The endogenous level of ADAMs in clinical specimens. (A)** The expression of ADAM10 and ADAM17 was detected in cancer (LSCC or LUAD) and paracancerous tissues, respectively. **(B)** Correlation analysis of N-Cadherin and ADAM10 in LSCC tissues.** (C)** Correlation analysis of survivin and ADAM10 in LSCC tissues.

The expression of ADAM10 or ADAM17 in LUAD specimens was examined by qPCR. As shown in Figure 1A, the expression of ADAM17 and ADAM10 in the selected 30 pairs of clinical specimens was higher in LUAD tissues than in adjacent tissues, and the expression of ADAM10 in LUAD tissues was significantly higher than that of ADAM17. As an important control, the expression of ADAM17 is much higher in LSCC compared with LUAD (Figure 1A). This indicated that of the two ADAMs, ADAM10 is the ADAM subtype that plays a major role in LUAD.

**Figure 2:**
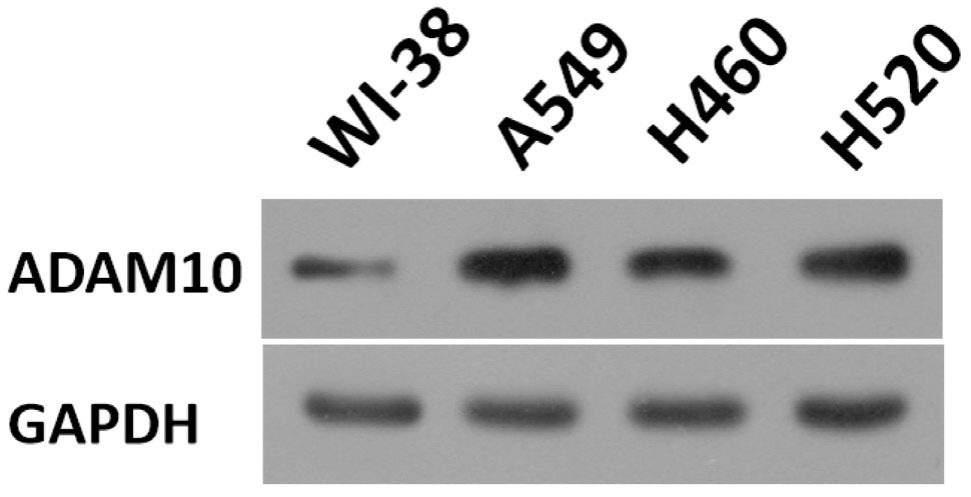
** The expression level of ADAM10 in lung derived cell lines.** The lung derived cell lines (WI-38 [a non-tumor cell line], A549 [a LUAD cell line], H460 [a LCC cell line], H520 [a LSCC cell line]) were cultured and harvested for western blot. The results were shown as images of western blot. GAPDH was used as the loading control.

To further reveal the role of ADAM10 in LUAD, a correlation between the expression of ADAM10 with the Notch pathway members was examined. As shown in Figure 1B and C, the expression of ADAM10 was positively correlated with the expression of N-cadherin or survivin, two typical downstream factors of the Notch pathway, in LUAD clinical specimens. Moreover, the expression level of ADAM10 in lung derived cell lines was also examined by western blot. As shown in Figure 2, the expression of ADAM10 in NSCLC cells (A549, H460 and H520) was much higher compared with its expression level in WI-38, a lung derived non-tumor cell line. Therefore, these findings suggested that ADAM10 participates in the activation of Notch pathway in LUAD.

### miR-140-3p targeting of ADMA10 in LUAD

**Figure 3:**
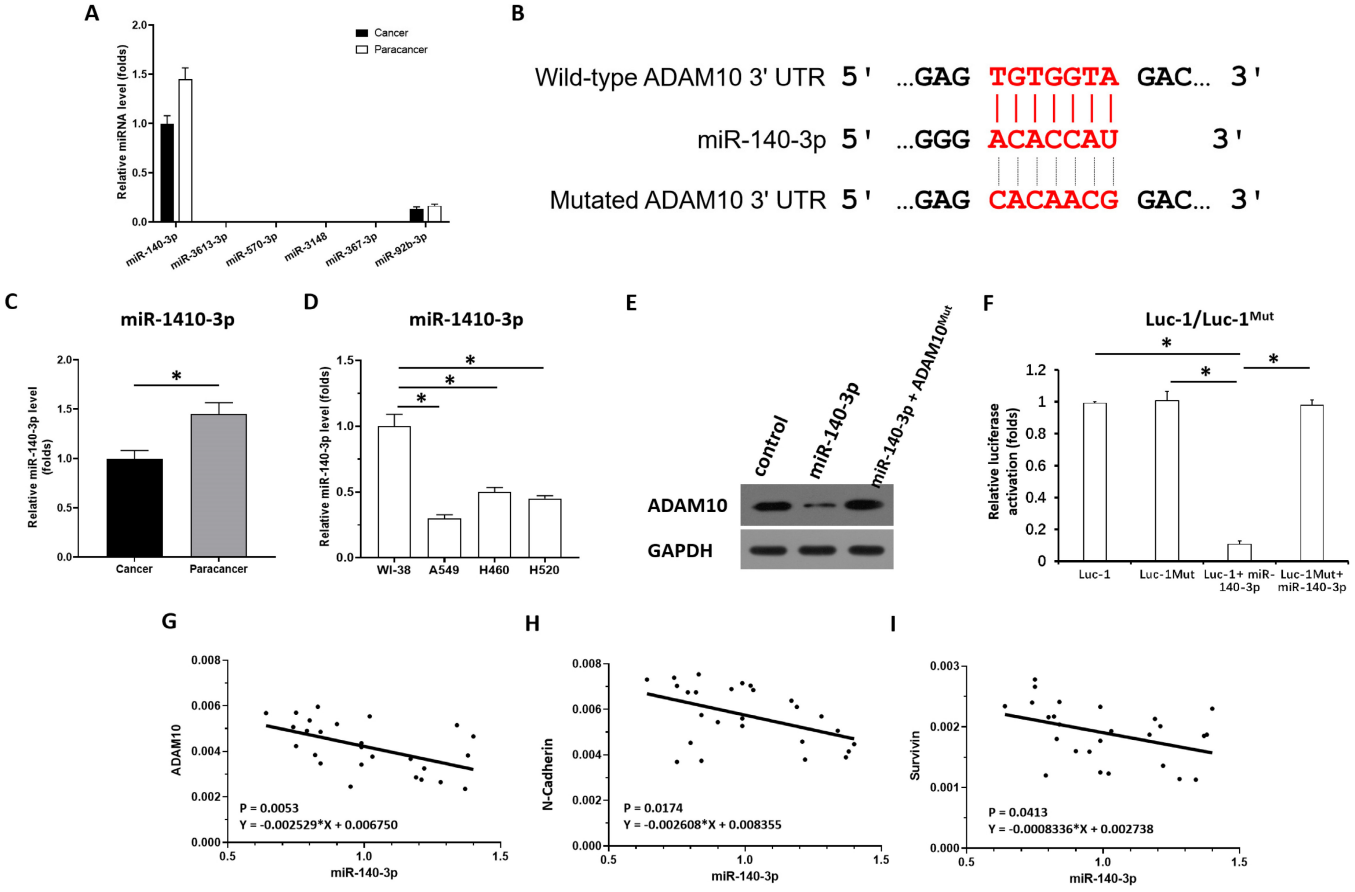
** miR-140-3p potentially targets the 3'UTR of ADAM10. (A)** The miRNAs (miR-140-3p, miR-3613-3p, miR-570-3p, miR-3148, miR-367-3p, miR-92b-3p) potentially targeting to ADAM10 with high scores was examined in the clinical specimens (the LUAD specimens and the paired non-tumor specimens) via qPCR. **(B)** The targeting sites of miR-140-3p in the 3'UTR of ADAM10. **(C)** The expression levels of miR-140-3p were detected in cancer (LUAD) and para-cancerous tissue. **(D)** The expression level of miR-140-3p in lung derived cell lines (WI-38 [a non-tumor cell line], A549 [a LUAD cell line], H460 [a LCC cell line], H520 [a LSCC cell line]) was examined by qPCR. **(E and F)** The effects of miR-140-3p on the expression of ADAM10 in A549 cells were confirmed by western blotting assays (E) or luciferase assyas (F). **(G)** Correlation analysis of miR-140-3p and ADAM10 expression in LSCC tissues. **(H)** Correlation analysis of miR-140-3p and N-cadherin expression in LSCC tissues. **(I)** Correlation analysis of miR-140-3p and surviving expression in LSCC tissues, *P<0.05.

To reveal the potential mechanisms associated with the high expression of AMAM10 in LUAD, the expression level of miRNAs which were predicted by online tool miRDB potentially targeting to ADAM10 was examined in clinical specimens. As shown in Figure 3A, among the miRNAs (miR-140-3p, miR-3613-3p, miR-570-3p, miR-3148, miR-367-3p, miR-92b-3p) potentially targeting to ADAM10 with high scores, the miR-140-3p was detected in clinical specimens (the LUAD specimens and the paired non-tumor specimens); whereas miR-3613-3p, miR-570-3p, miR-367-3p, miR-3148, could not be detected in clinical specimens. Although miR-92b-3p can be detected in clinical specifications, its expression level is far lower than miR-140-3p. Therefore, miR-140-3p may be a miRNA with physiological and clinical significance that targets on ADAM10 in LUAD.

Next, as shown in Figure 3B and C, miR-140-3p could target the 3'UTR of ADAM10 in LUAD. The expression of miR-140-3p was much lower in LUAD specimens compared with the paired non-tumor tissues (Figure 3C). Moreover, the expression level of miR-140-3p in lung derived cell lines was also examined by qPCR. As shown in Figure 3D, the expression of miR-140-3p in NSCLC cells (A549, H460 and H520) was much lower compared with its expression level in WI-38, a lung derived non-tumor cell line. Overexpression of miR-140-3p downregulated the protein levels of wild-type ADAM10 (the endogenous ADAM10 expressed in A549 cells) but not the ADAM10 harboring a mutated miR-140-3p target site in the 3'UTR (ADAM10^Mut^) (Figure 3E) in A549 cells. Thus, miR-140-3p repressed the expression of ADAM10 in A549 cells. The effect of miR-140-3p on ADAM10 was further confirmed by the luciferase assays (Figure 3F). Moreover, the correlation between the expression of miR-140-3p and the Notch pathway was examined. As shown in Figure 3G, the expression of miR-140-3p was negatively associated with the expression of ADAM10 in LUAD clinical specimens. The expression of miR-140-3p was also negatively associated with the expression of N-cadherin or survivin, two typical downstream factors of the Notch pathway, in LUAD clinical specimens (Figure 3H and 3I). Therefore, these findings suggested that miR-140-3p could inhibit the expression of ADAM10 protein by targeting the 3'UTR of ADAM10 in LUAD.

### miR-140-3p repressed the activation of the Notch pathway

**Figure 4:**
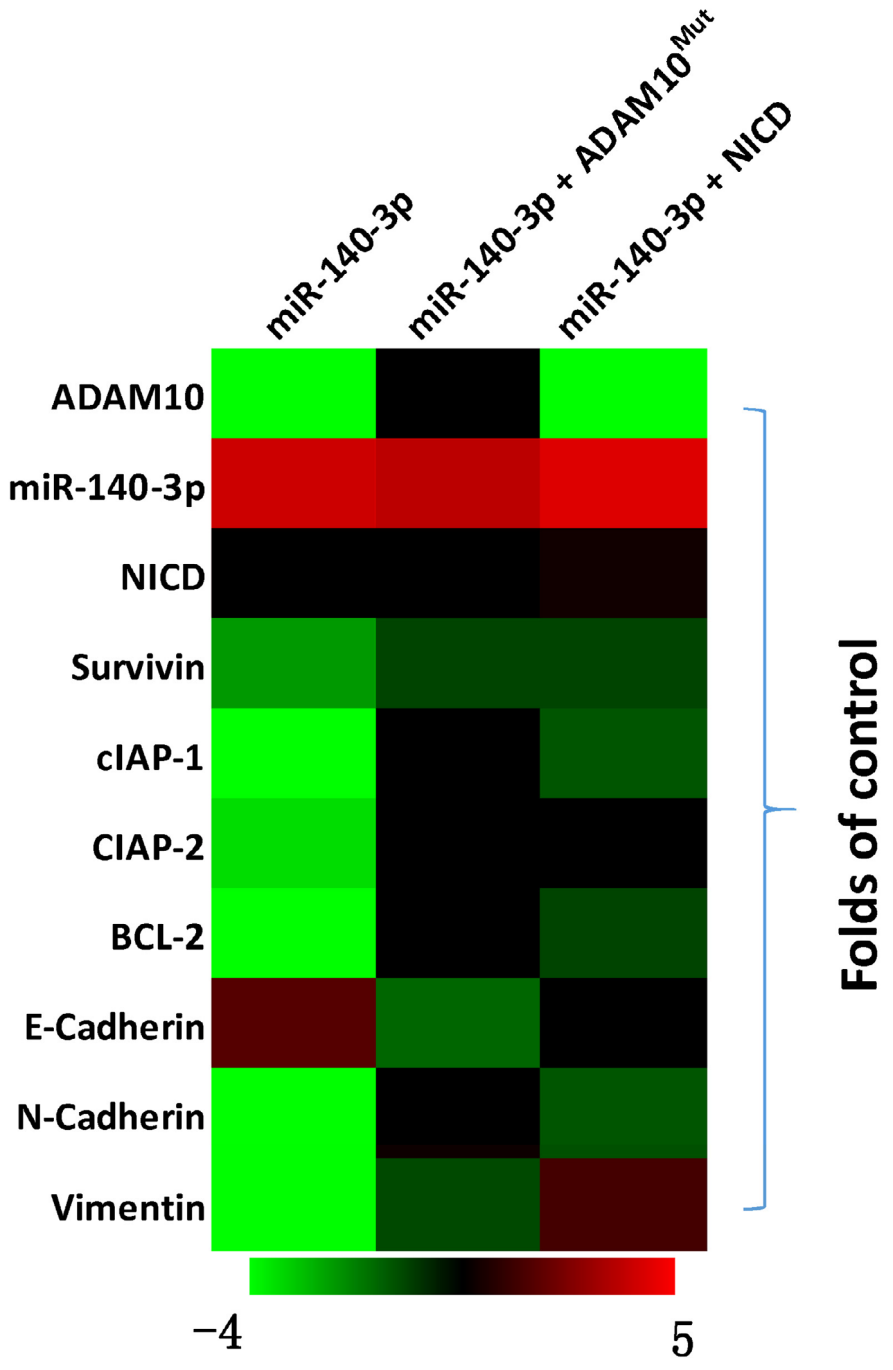
The effects of miR-140-3p on the Notch pathway in A549 cells. A549 cells were transfected with vectors and harvested for qPCR experiments. The related expression level (fold-change with respect to the control group) of miR-140-3p (hsa-pre-miR-140), AMDA10 or Notch pathway related factors are shown as a heat-map.

The above results indicated that miR-140-3p inhibited the expression of ADAM10 in A549 cells. To further examine the effects of miR-140-3p on the Notch pathway, the expression of Notch pathway's downstream factors was examined by the qPCR. As shown in Figure 4, overexpression of miR-140-3p in A549 cells repressed the expression of Notch pathway downstream anti-apoptosis or the pro-survival related factors (e.g., survivin, cIAPs) and EMT related factors (e.g., N-cadherin, vimentin). Co-transfection of A549 cells with miR-140-3p with ADAM10^Mut^ or NICD almost completely abolished the inhibitory effects of miR-140-3p on the Notch pathway, while NICD did not influence the expression of ADAM10 in A549 cells. Therefore, miR-140-3p repressed the activation of Notch pathway by targeting to the 3'UTR of ADAM10 in A549 cells.

**Figure 5:**
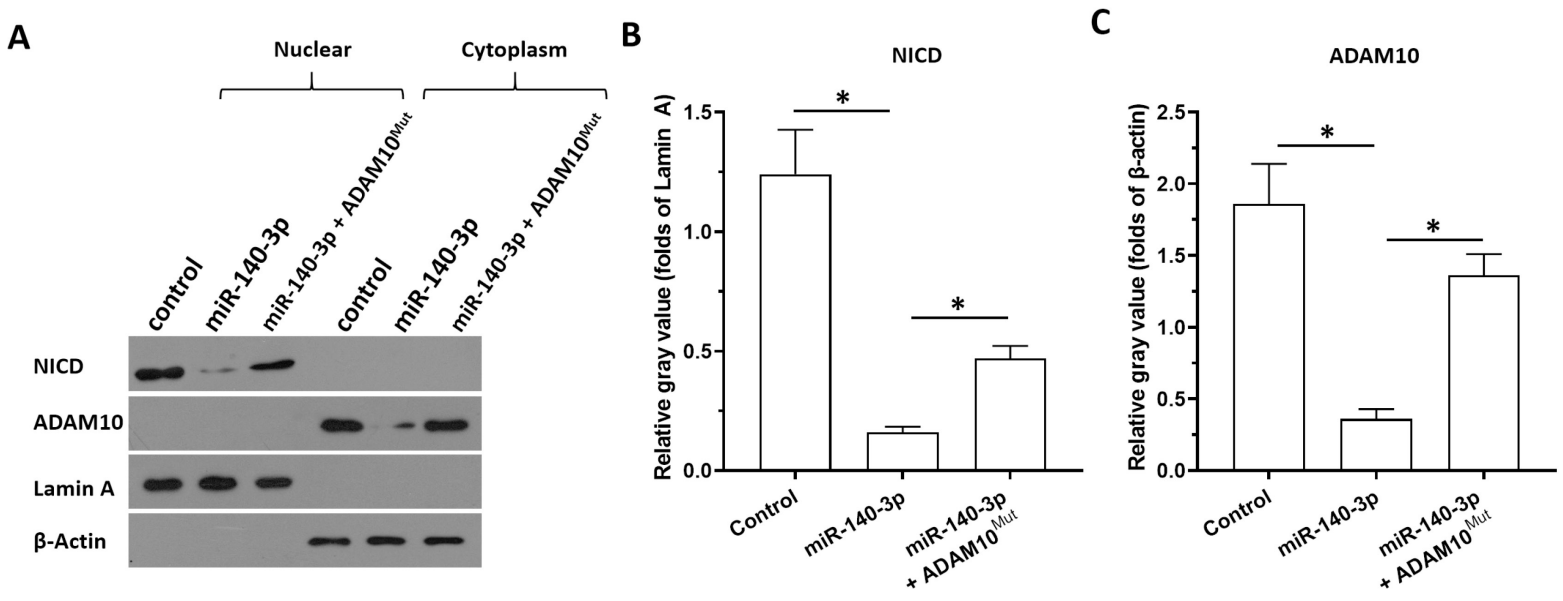
** The specificity of miR-140-3p on the Notch pathway.** The effects of miR-140-3p on the cleavage of Notch protein shown in cellular sub-fraction assays using A549 cells. The accumulation of NICD in the nuclear sub-fraction of A549 cells reflected the cleavage of Notch protein. Lamin A was used as the indicator of nuclear proteins; whereas the β-Actin was used as the indicator of cytoplasmic proteins. The results were shown as the images of western blot **(A)** or the quantitative results** (B and C)**. *P<0.05.

Next, the specificity of miR-140-3p on the ADAM10/Notch pathway was further examined using cellular sub-fraction assays. The cleaving of Notch protein was directly reflected by the accumulation of NICD in nuclear sub-fraction of A549 cells. As shown in Figure 5, ADAM10 was detected in the cytoplasm sub-fraction and the NICD was detected in the nuclear sub-fraction of A549 cells. Overexpression of miR-140-3p decreased the expression of ADAM10 in the cytoplasmic fraction and decreased the accumulation of NICD in the nuclear fraction of A549 cells. Therefore, miR-140-3p inhibited the cleavage of Notch protein and led to the nuclear accumulation of NICD to inhibit the activation of Notch pathway.

### miR-140-3p enhanced the sensitivity of AC cells to antitumor agents

**Figure 6:**
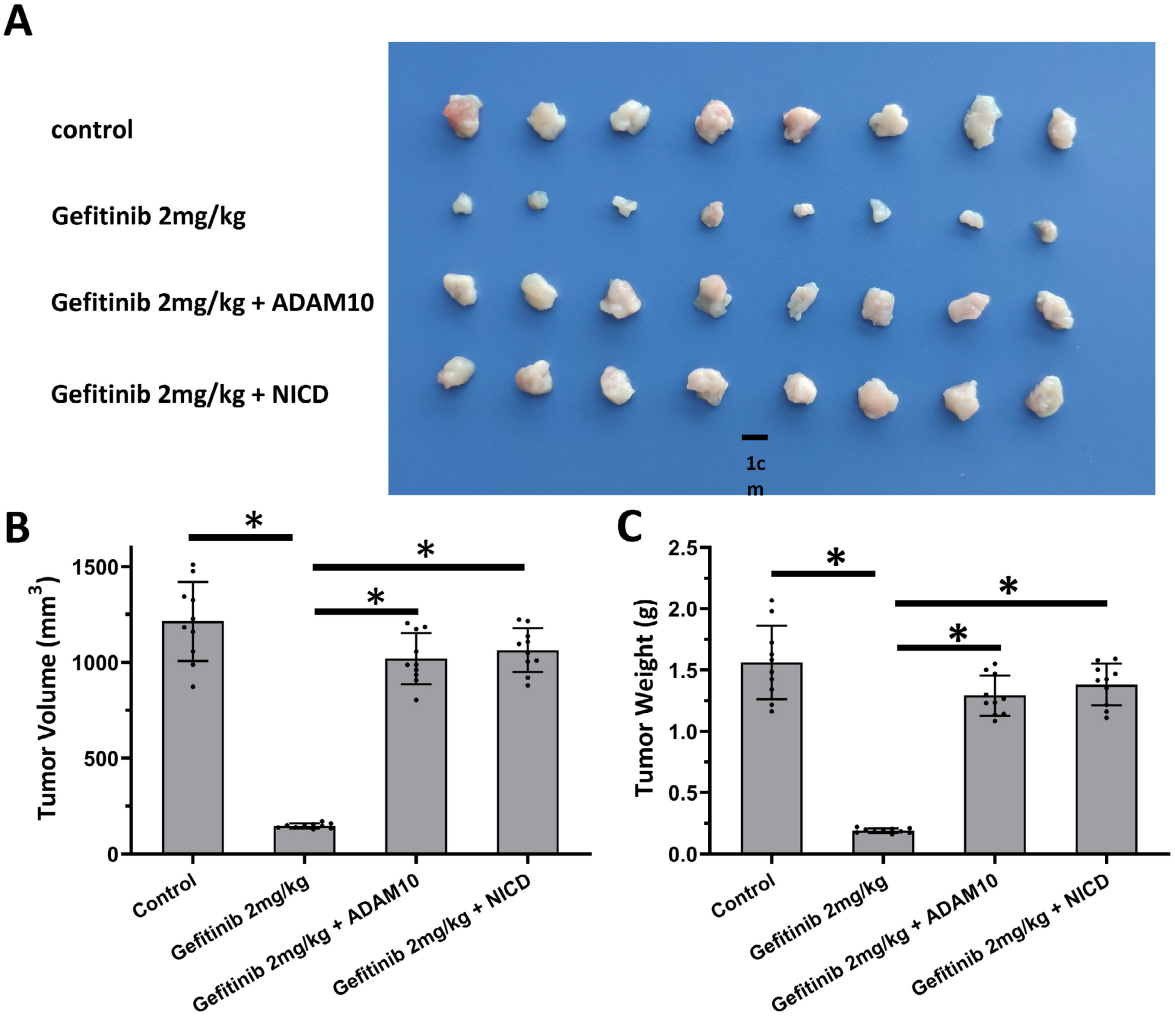
** Activation of Notch pathway by Notch protein's cleavage enhanced the resistance of A549 cells.** A549 cells which were transfected with expression vectors (control, NICD or ADAM10) were injected into the nude mice to form the subcutaneous tumor tissues. Mice received 2mg/kg dose of gefitinib via oral administration. The results shown are images of tumor tissues **(A)**, the tumor volumes **(B)**, and the tumor weights **(C)**. *P<0.05.

The above results revealed the effects of miR-140-3p on the ADAM10/Notch pathway. To confirm the role of ADAM10/Notch pathway, MTT and the nude mice model was used. As shown in Table 3, overexpression of ADAM10 or NICD enhanced the resistance of A549 cells to antitumor agents, and the IC_50_ values of these antitumor agents on A549 were increased. Overexpression of NICD or ADAM10 also enhanced the resistance of A549 cells to gefitinib, a typical molecular targeted agent used for LUAD treatment, in the nude mice model (Figure 6). These results confirmed the roles of ADAM10/Notch in LUAD cells.

To further examine the effects of miR-140-3p on LUAD's sensitivity to antitumor drugs, the MTT assays were performed. As shown in Table 3, the antitumor agents, including the cytotoxic chemotherapies (gemcitabine, paclitaxel, etoposide or doxorubicin) or the molecular targeted agents (gefitinib, erlotinib, osimertinib or anlotinib) inhibited the survival of A549 cells in a dose-dependent manner. Overexpression of miR-140-3p enhanced the sensitivity of A549 cells to these agents: the IC_50_ values of these agents in A549 cells decreased in the presence of miR-140-3p (Table 3). Transfection of AMAM10^Mut^ or NICD repressed the effects of miR-140-3p on the sensitivity of A549 cells to the antitumor agents (Table 3). To further confirm the effect of miR-140-3p on LUAD cells, H460 (LCC cell line) and H520 (LSCC cell line), two typical cell lines of the other two types of NSCLC were used. Overexpression of miR-140-3p enhanced the sensitivity of H460 (Table 4) or H520 (Table 5) cells to these agents: the IC_50_ values of these agents in (Table 4) or H520 (Table 5) cells decreased in the presence of miR-140-3p. Transfection of AMAM10^Mut^ or NICD repressed the effects of miR-140-3p on the sensitivity of (Table 4) or H520 (Table 5) cells to the antitumor agents. Therefore, miR-140-3p enhanced the sensitivity of A549 cells to antitumor agents by targeting ADAM10.

**Figure 7:**
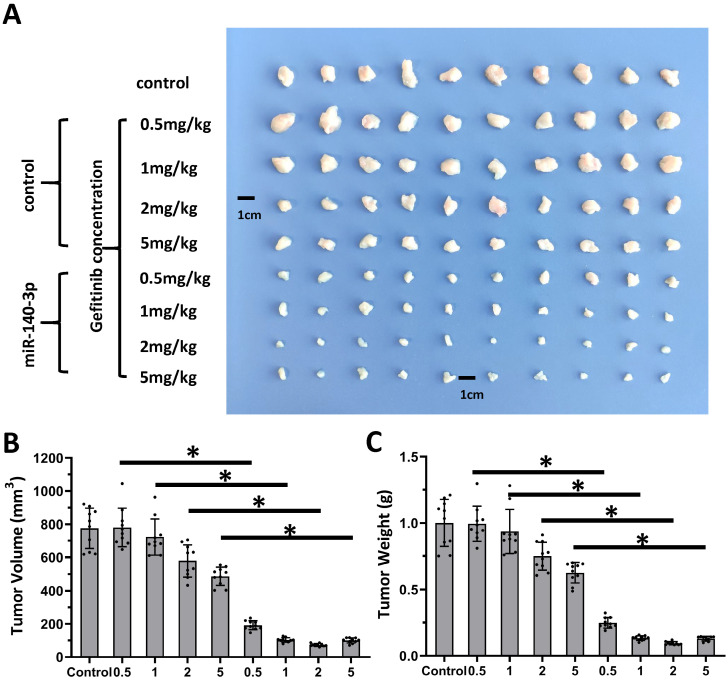
** Overexpression of miR-140-3p enhanced the sensitivity A549 cells to Gefitinib.** A549 cells which were transfected with vectors (control or miR-140-3p) were injected into the nude mice to form the subcutaneous tumor tissues. Mice received the indicated doses of gefitinib via oral administration. The results are shown as images of tumor tissues **(A)**, the tumor volumes** (B)**, and the tumor weights** (C)**. *P<0.05.

**Figure 8:**
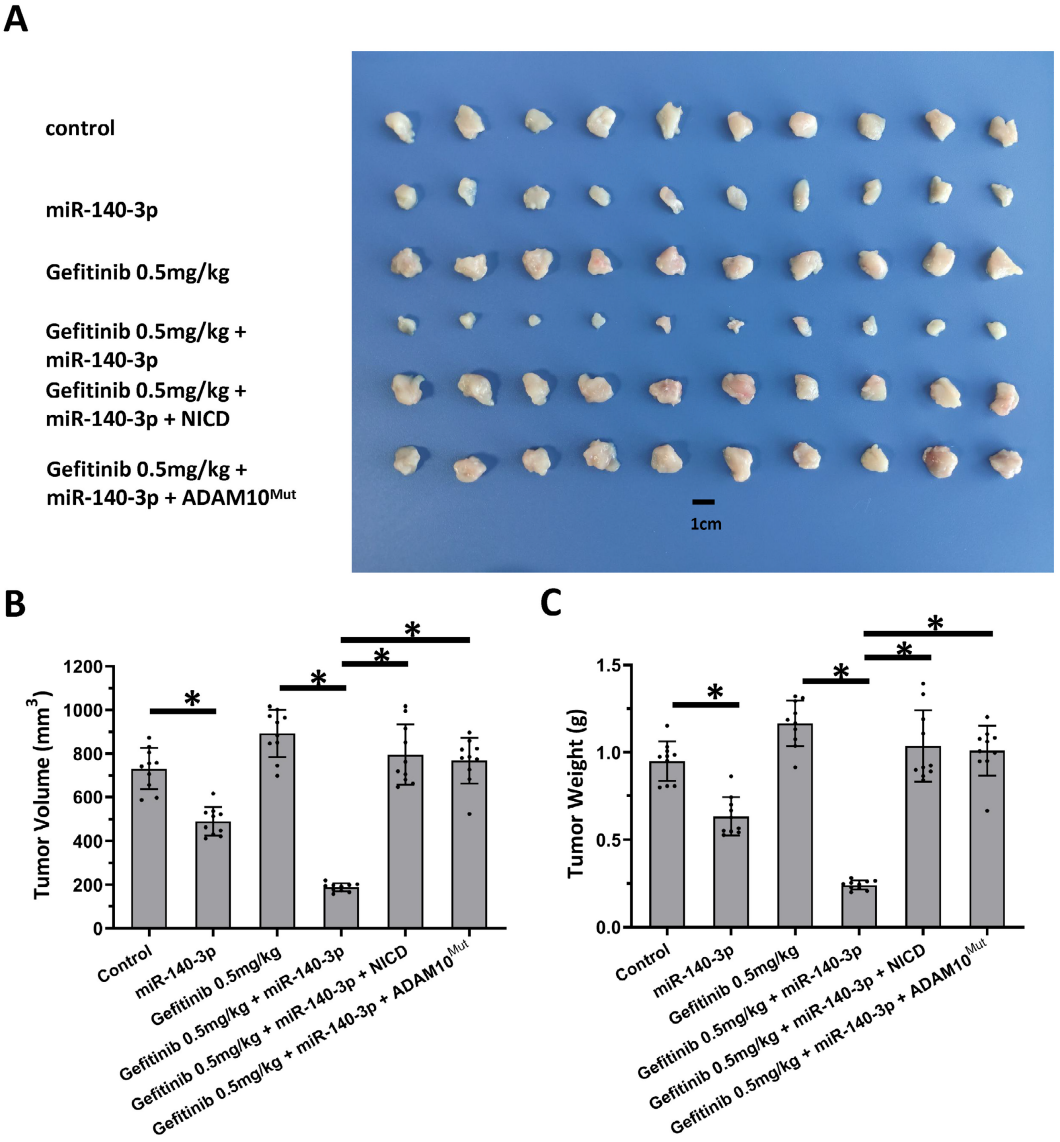
** Overexpression of miR-140-3p enhanced the sensitivity A549 cells to Gefitinib.** A549 cells which were transfected with vectors (control, miR-140-3p, miR-140-3p + NICD or miR-140-3p + ADAM10_Mut_) were injected into nude mice to form the subcutaneous tumor tissues. Mice received the indicated 0.5 mg/kg dose of gefitinib via oral administration. The results are shown as images of tumor tissues** (A)**, tumor volumes **(B)** and tumor weights **(C)**. *P<0.05.

**Figure 9:**
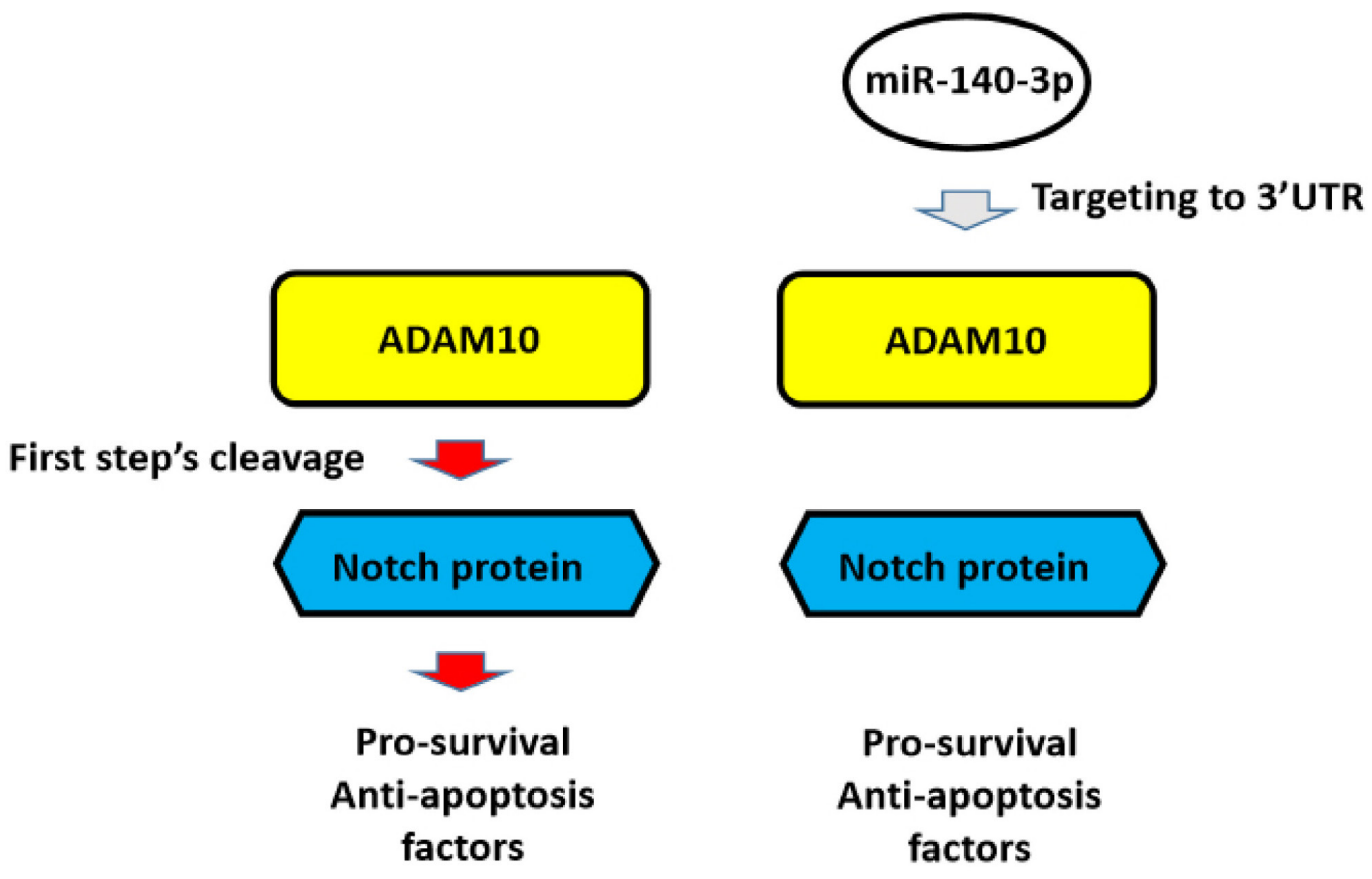
** The proposal model of miR-140-3p and its downstream pathway in LUAD in the present work.** In A549 cells, Notch protein can be cleaved by ADAM10 and the NICD of Notch protein translocated into the nuclear to mediate the pro-survival or anti-apoptosis rated factors' expression. These factors participate in the resistance of A549 cells to antitumor drugs. Overexpression of miR-140-3p inhibited the expression of ADAM10 by targeting to ADAM10's 3'UTR and in turn inhibited the activation of Notch pathway via inhibiting the cleavage of Notch protein by ADAM10.

Next, the nude mice model was used in combination with gefitinib chosen as the representative drugs. As shown in Figure 7, oral administration of gefitinib inhibited the subcutaneous growth of A549 cells in a dose-dependent manner. Overexpression of miR-140-3p enhanced the *in vivo* antitumor activation of gefitinib on A549 cells. Moreover, as shown in Figure 8, overexpression of miR-140-3p in A549 cells exerted a certain anti-tumor activity, and gefitinib at a dose of 0.5 mg/kg was selected. Gefitinib at this dose had no significant anti-tumor activity (Figure 8). However, exposure to miR-140-3p significantly up-regulated the antitumor activity of 0.5 mg/kg gefitinib (Figure 8). Thus, the simultaneous transfection of NICD or ADAM10^Mut^ inhibited the effects of miR-140-3p (Figure 8). These results indicated that miR-140-3p enhanced the *in vitro* and *in vivo* antitumor activation of antitumor agents by targeting the 3'UTR of ADAM10 in A549 cells (Figure 9).

### The compensatory role of ADAM17 and ADAM10 in inducing LUAD cells to resist tumor drug resistance

The above results mainly focused on the effects of miR-140-3p on the ADAM10/Notch pathway. To confirm the role of ADAM10/Notch pathway and the compensatory role of ADAM17 and ADAM10 in inducing LUAD cells to resist tumor drug resistance, MTT method was used. As shown in Table 6, Inhibition of ADAM17 expression by siRNA can increase the sensitivity of A549 cells to anti-tumor drugs; ADAM10 was overexpressed in A549 cells. It can completely inhibit the effect of ADAM17. Similarly, inhibition of ADAM10 expression by siRNA could increase the sensitivity of A549 cells to antitumor drugs; ADAM17 was overexpressed in A549 cells. It can completely inhibit the effect of ADAM10. These results can reflect the compensatory role of ADAM10-ADAM17 in the anti-cancer drug resistance of A549 cells.

## Discussion

The Notch protein plays a central role in the Notch pathway. There are four sub-types of Notch protein (Notch receptors): NOTCH-1, NOTCH-2, NOTCH-3, and NOTCH-4 characterized by the extra-cellular domains and intracellular domains 54. Among these four types of Notch receptors, the NICD is highly conserved, however, the Notch extracellular domains (EICD) of the four types of Notch receptor shows high sequence variation 55. Therefore, in this study, transfecting the expression vector for NICD in A549 cells, or detecting the expression of NICD in the nucleus in the cellular sub-fraction experiment, could avoid the compensatory effect between the four Notch proteins. The expression of NICD was visually observed by the effect of Adam10 on the first step of cleavage of NOTCH proteins in the nucleus. Using miRNA to lower the expression of ADAM10 inhibited the NOTCH pathway, and ultimately up-regulated the sensitivity of malignant tumor cells against tumor drugs. Yang et al.2019 showed that repressing the expression of ADAM17 in HCC cells by miR-3163 enhanced the sensitivity of HCC cells to molecular targeted agents 28. Conversely, studies investigating γ-secretase, which mediates the second step of Notch cleavage is also of great significance 25. The results of Zhao et al. 2021 showed that miR-27-3p can act on the catalytic subunit PSEN-1 of γ-secretase to inhibit the cleavage of Notch, and ultimately up-regulate the sensitivity of triple-negative breast cancer (TNBC) cells to Olaparib 25. Studies by Jia et al. 2021 reviewed the small molecule inhibitors that act on γ-secretase in detail 26,56. γ-secretase has complex structure consisting of an intramembrane aspartate-lyase with multi-subunits, including the PSEN-1, the nicastrin subunit (NCSTN), the anterior pharynx-defective subunit (APH-1), and the presenilin enhancer subunit (PEN-2) 26,56. When targeting PSEN1 alone, the effects of other subunits on PSEN-1 also need to be considered.

Currently, an important part of the anti-tumor treatment strategy of NSCLC, especially LUAD, is treatment with a variety of anti-tumor drugs—mainly various cytotoxic chemotherapeutics and molecular targeted drugs as protein kinase inhibitors or the combined use of these drugs 57,58. The present study investigated four cytotoxic chemotherapeutic agents and four molecular targeted drugs. Overexpression of miR-140-3p in A549 cells up-regulated the anti-tumor activity of the selected drugs by acting on ADAM10. These findings were essentially consistent with our expectations: the activation of the Notch pathway up-regulates the resistance of cells to anti-tumor treatment strategies, so there should be no selective resistance to cytotoxic drugs or molecular targeted drugs. On this basis, the four selected cytotoxic chemotherapy drugs, gemcitabine, paclitaxel, etoposide or doxorubicin, all act on biological macromolecules such as DNA and microtubules, and kill malignant tumor cells by causing macromolecular damage 59-62. Among the selected four molecularly-targeted drugs, gefitinib, erlotinib and osimertinib, all mainly act on the EGFR, while anlotinib is a multi-target protein kinase inhibitor that targets the VEGFR 63,64. Gefitinib is considered the first-generation of small molecule protein kinase inhibitors acting on EGFR, while erlotinib and osimertinib are the second- and third-generation agents acting on small molecule protein kinase inhibitors 63,64; all these drugs were used in this study and killed A549 cells in a dose-dependent manner. These drugs mainly act on the EGFR and the difference in their activity is mainly determined by the higher mutational status of the EGFR 64. In the future, it may be of great significance to detect EGFR mutations in clinical specimens and prepare corresponding patients derived cells (PDCs). It is worth mentioning that traditional molecular targeted drugs, as represented by Gefitinib, all act on the EGFR, and related research and development strategies should continue to exploit this potential. Currently, new molecular targeted drugs, represented by anlotinib act on VEGFR-related targets 63,64, have been approved for marketing for NSCLC treatment, which will provide additional therapeutic options for NSCLC patients.

In addition to AC, NSCLC also includes LSCC 65. At present, the research on LUAD is relatively in-depth, while studies on LSCC are relatively lagging behind, with several reports on LUAD-related molecular targeted drugs but few studies on LSCC 51. Zhou et al. reported that VEGFR and other anlotinib targets are widely expressed in LSCC and their expression is not significantly different from the expression in LUAD 51, in addition, anlotinib can also effectively kill LSCC cells 51. This study focuses on A549, the most common and recognized cell line model of LUAD, although, we will study the role of ADAMs in LSCC further in the future. The results of this study show that ADAM10 plays a major role in LUAD among AMAMs, while ADAM17 may play a major role in LSCC. It is worth mentioning that Qingchun Zhao's research group used a research strategy of old drugs for new applications, and found that terfenadine can act as a small molecule inhibitor of ADAM17, and can inhibit the activity of the ADAM17/Notch pathway in NSCLC cells, ultimately overcoming NSCLC cells resistance and improve sensitivity to oncology drugs 26,37,66-69. Similar to terfenadine, ZIDI-8, a superior compound, was discovered through structural optimization and transformation 26,37,66-69. Since ADAM17 is not expressed at a lower level than ADAM10 in LUAD cells, an effect of these drugs on ADAM10 cannot be ruled out. In addition to LSCC and LUAD, NSCLC also includes large cell lung cancer, which also merits research attention. In the future, the expression level of ADAMs will also be tested in tissue specimens and cell lines of large cell lung cancer.

The miRNA-140-3p has been considered as an important regulator of NSCLC cells. Wan et al., 2021 reported that miRNA-140-3p represses the proliferation of lung adenocarcinoma cells by targeting thymidylate synthetase 70; Hu et al., 2022 also reported that miR-140-3p inhibits the progression of NSCLC by targeting Janus kinase 1 71. One miRNA may have multiple targets, so it plays a variety of physiological functions 72-74. This makes the results of miR-140-3p targeting on other targets reported in these articles do not affect the finding that miR-140-3p can target on ADAM10 in this study. Our study focused on the expression level of ADAM17 and ADAM10 in lung adenocarcinoma related specimens and the expression level of miRNA acting on ADAM10, and finally found that miR-140-3p in lung adenocarcinoma tissues has more clinical significance compared with other miRNAs. This study explored and preliminarily clarified that ADAM10 may play a major role in NSCLC compared with ADAM17, and miR-140-3p is not only one of the possible mechanisms of ADAM10 overexpression in NSCLC, but also can potentially be used as a new therapeutic strategy targeting ADAM10. The miR-140-3p could modulate the sensitivity of NSCLC cells by targeting other proteins. Wu et al., 2020 also showed that miR-140-3p could enhance the sensitivity of NSCLC cells to cisplatin by targeting Wnt/β-catenin signaling to attenuate the stem cell-like properties 75. It is worth mentioning that this study mainly focuses on the downstream pathway of miR-140-3p (ADAM10/Notch), and the upstream signal pathway of miR-140-3p is also worth studying, and there have been some research reported: Wei et al., 2022 reported that Circ_0020123 enhances the cisplatin resistance in NSCLC cells by sponging miR-140-3p/HOXB5 76; Wang et al., 2022 revealed that Hypoxia-induced PVT1 promotes lung cancer chemoresistance by PVT1/miR-140-3p/ATG5 axis 77. Such research is of great significance to better understand the molecular mechanism of miR-140-3p. The methylation analysis of miR-140-3p promoter region can also be attempted 78,79.

## Figures and Tables

**Table 1:** The baseline information of patients involved in the present work

Characters	Values
Age	55 (38~75) (Median [upper and lower limit])
Gender	Male (58%, 15/26)
Tumor stage	I (65%, 17/26) (IA and IB)
	II (35%, 9/26) (IIA and IIB)
Tumor number	1 (92%, 24/26)
	2 (8%, 2/26)
Tumor sites	Left lower lobe (11%, 3/26)
	Left upper lobe (27%, 7/26)
	Right lower lobe (19%, 5/26)
	Right middle lobe (-)
	Right upper lobe (43%, 11/26)
Tumor location	Central (15%, 8/26)
	Peripheral (85%, 22/26)
Tumor sizes	1.4 (0.4~2.2 [cm]) (Median [upper and lower limit])

**Table 2:** The concentrations of antitumor agents in the present work

Agents	Gemcitabine	Paclitaxel	Etoposide	Doxorubicin	Gefitinib	Erlotinib	Osimertinib	Anlotinib
DMSO (mmol/L)	3	0.3	3	1	10	10	10	10
	1	0.1	1	0.3	3	3	3	3
	0.3	0.03	0.3	0.1	1	1	1	1
	0.1	0.01	0.1	0.03	0.3	0.3	0.3	0.3
	0.03	0.03	0.03	0.01	0.1	0.1	0.1	0.1
	0.01	0.01	0.01	0.003	0.03	0.03	0.03	0.03
	0.003	0.003	0.003	0.001	0.01	0.01	0.01	0.01
DMEM (μmol/L)	3	0.3	3	1	10	10	10	10
	1	0.1	1	0.3	3	3	3	3
	0.3	0.03	0.3	0.1	1	1	1	1
	0.1	0.01	0.1	0.03	0.3	0.3	0.3	0.3
	0.03	0.03	0.03	0.01	0.1	0.1	0.1	0.1
	0.01	0.01	0.01	0.003	0.03	0.03	0.03	0.03
	0.003	0.003	0.003	0.001	0.01	0.01	0.01	0.01

**Table 3:** Overexpression of NIVD or ADAM10 enhanced the resistance of LUAD cells to antitumor agents

Agents	control	NICD	ADAM10
	IC_50_ values (μmol/L)		
Gemcitabine	0.30±0.12	1.77±0.87	1.66±0.28
Paclitaxel	0.10±0.01	0.62±0.37	0.59±0.66
Etoposide	0.44±0.05	2.34±0.48	1.94±0.80
Doxorubicin	0.29±0.04	1.62±0.43	1.81±0.41
Gefitinib	1.79±0.35	10.68±0.31	9.94±1.11
Erlotinib	1.55±0.94	9.08±0.74	10.42±0.63
Osimertinib	1.08±0.47	12.28±1.01	11.03±0.95
Anlotinib	0.92±0.55	10.49±0.72	8.06±1.33

Table notes: NICD, the intra-cellular domain of Notch protein; AMDA10, A disintegrin and metalloproteinase domain 10.

**Table 4:** miR-140-3p enhanced the sensitivity of LUAD cells A549 to antitumor agents by targeting to the 3'UTR of ADAM10

Agents	control	miR-140-3p	miR-140-3p + AMDA10^Mut^	miR-140-3p + NICD
	IC_50_ values (μmol/L)			
Gemcitabine	0.42±0.15	0.07±0.01	0.59±0.10	0.50±0.11
Paclitaxel	0.11±0.01	0.03±0.00	0.15±0.01	0.13±0.00
Etoposide	0.45±0.10	0.12±0.06	0.50±0.07	0.57±0.34
Doxorubicin	0.37±0.22	0.06±0.02	0.48±0.20	0.44±0.06
Gefitinib	1.99±0.65	0.61±0.23	2.21±0.78	2.41±0.87
Erlotinib	1.74±0.29	0.36±0.08	1.80±0.86	1.65±0.26
Osimertinib	1.22±0.62	0.25±0.05	1.24±0.23	1.00±0.32
Anlotinib	1.05±0.30	0.14±0.03	0.90±0.21	1.37±0.25

Table notes: NICD, the intra-cellular domain of Notch protein; AMDA10^Mut^, A disintegrin and metalloproteinase domain 10 with mutated miR-140-3p's targeting sites in 3'UTR.

**Table 5:** miR-140-3p enhanced the sensitivity of LCC cells H460 to antitumor agents by targeting to the 3'UTR of ADAM10

Agents	control	miR-140-3p	miR-140-3p + AMDA10^Mut^	miR-140-3p + NICD
	IC_50_ values (μmol/L)			
Gemcitabine	0.35±0.25	0.06±0.02	0.50±0.08	0.38±0.29
Paclitaxel	0.23±0.05	0.01±0.00	0.20±0.00	0.25±0.26
Etoposide	0.46±0.19	0.06±0.03	0.44±0.16	0.40±0.13
Doxorubicin	0.30±0.08	0.12±0.04	0.30±0.05	0.35±0.11
Gefitinib	2.35±0.21	1.03±0.56	2.15±0.68	1.56±0.70
Erlotinib	1.87±0.45	0.65±0.48	1.91±0.41	1.95±0.75
Osimertinib	1.50±0.42	0.32±0.07	1.73±0.21	1.61±0.18
Anlotinib	1.23±0.16	0.35±0.24	1.00±0.62	0.98±0.24

Table notes: NICD, the intra-cellular domain of Notch protein; AMDA10^Mut^, A disintegrin and metalloproteinase domain 10 with mutated miR-140-3p's targeting sites in 3'UTR.

**Table 6:** miR-140-3p enhanced the sensitivity of LSCC cells H520 to antitumor agents by targeting to the 3'UTR of ADAM10

Agents	control	miR-140-3p	miR-140-3p + AMDA10^Mut^	miR-140-3p + NICD
	IC_50_ values (μmol/L)			
Gemcitabine	0.32±0.08	0.08±0.01	0.34±0.10	0.56±0.44
Paclitaxel	0.15±0.06	0.04±0.01	0.11±0.00	0.16±0.08
Etoposide	0.69±0.45	0.10±0.00	0.50±0.04	0.57±0.10
Doxorubicin	0.41±0.25	0.05±0.03	0.37±0.12	0.31±0.03
Gefitinib	1.77±0.39	0.76±0.20	1.60±0.58	1.74±0.21
Erlotinib	1.66±0.33	0.26±0.12	1.10±0.39	1.85±0.17
Osimertinib	1.65±0.51	0.18±0.11	1.77±0.63	1.75±0.43
Anlotinib	1.24±0.30	0.17±0.09	1.06±0.86	1.21±0.75

Table notes: NICD, the intra-cellular domain of Notch protein; AMDA10^Mut^, A disintegrin and metalloproteinase domain 10 with mutated miR-140-3p's targeting sites in 3'UTR.

**Table 7:** There is a compensatory effect between ADAM10 and ADAM17 in the induction of anti-tumor drug resistance in A549 cells

Agents	control	siADAM17	siADAM10	siADAM17+ADAM10	siADAM10+ADAM17
	IC_50_ values (μmol/L)				
Gemcitabine	0.50±0.27	0.31±0.14	0.11±0.00	0.56±0.31	0.45±0.13
Paclitaxel	0.15±0.02	0.07±0.02	0.02±0.00	0.24±0.09	0.34±0.15
Etoposide	0.42±0.06	0.18±0.05	0.06±0.01	0.58±0.21	0.58±0.27
Doxorubicin	0.49±0.24	0.23±0.10	0.13±0.07	0.52±0.30	0.63±0.25
Gefitinib	2.15±0.32	0.71±0.54	0.38±0.10	2.40±0.96	1.94±0.77
Erlotinib	1.80±0.43	0.97±0.46	0.40±0.26	1.97±0.52	1.68±0.73
Osimertinib	1.33±0.67	0.59±0.12	0.25±0.09	1.66±0.85	1.86±0.33
Anlotinib	1.29±0.63	0.48±0.09	0.21±0.05	1.35±0.91	1.51±0.64

Table notes: NICD, the intra-cellular domain of Notch protein; AMDA10^Mut^, A disintegrin and metalloproteinase domain 10 with mutated miR-140-3p's targeting sites in 3'UTR.
